# An enhanced algorithm for multiple sequence alignment of protein sequences using genetic algorithm

**DOI:** 10.17179/excli2015-302

**Published:** 2015-12-15

**Authors:** Manish Kumar

**Affiliations:** 1 Department of Computer Science and Engineering, Indian School of Mines, Dhanbad, Jharkhand, India

**Keywords:** bioinformatics, multiple sequence alignment, genetic algorithm, crossover operator, mutation operator

## Abstract

One of the most fundamental operations in biological sequence analysis is multiple sequence alignment (MSA). The basic of multiple sequence alignment problems is to determine the most biologically plausible alignments of protein or DNA sequences. In this paper, an alignment method using genetic algorithm for multiple sequence alignment has been proposed. Two different genetic operators mainly crossover and mutation were defined and implemented with the proposed method in order to know the population evolution and quality of the sequence aligned. The proposed method is assessed with protein benchmark dataset, e.g., BALIBASE, by comparing the obtained results to those obtained with other alignment algorithms, e.g., SAGA, RBT-GA, PRRP, HMMT, SB-PIMA, CLUSTALX, CLUSTAL W, DIALIGN and PILEUP8 etc. Experiments on a wide range of data have shown that the proposed algorithm is much better (it terms of score) than previously proposed algorithms in its ability to achieve high alignment quality.

## Introduction

The sequence alignment of three or more biological sequences such as the Protein, DNA or RNA (Auyeung and Melcher, 2005[3]; Wei et al., 2013[66]) is known as the multiple sequence alignment (Hamidi et al., 2013[21]). One of the standard techniques in bioinformatics for reviling the relationship between collections of evolutionarily or structurally related protein is sequence alignment. 

Sequence alignment are extensively be used for improving the secondary and tertiary structure of protein and RNA sequences, which is used for drug designing and also to find distance between organism. In MSA, the foremost effort is made to find the optimal alignment for a group of biological sequences. In the past research, we have observed several reliable and efficient techniques for alignment of multiple sequences, which includes evolutionary algorithm (GA) (Peng et al., 2011[51]), HMM (Eddy, 1998[13]) and the generic probabilistic metaheuristic for the global optimization problem (Kirkpatrick et al., 1983[32]). 

One of the widely studied branches in bioinformatics is sequence similarity, also known as a subset of sequence analysis. The available molecular sequence data have enough resources that can teach us about the structure, function and evolution of biological macromolecules. The main objective of an MSA is to align sequences which can show the biological relationship between the input sequences, but to develop a reliable MSA program is never easy. In general the MSA problem can be seen as: Let N number of sequences is supplied as input with a predetermined scoring scheme for finding the best matches among the letters (as every sequences consists of a series of letter). Although, definition stated here is simple but still it requires certain input such as the selection of input sequence and comparison model along with the optimization of the model to get completed in all respect. There are various issues demonstrated in the literature (Aniba et al., 2010[1]; Pop and Salzberg, 2008[53]; Sellers,1984[55]) for alignment of protein sequences. First, the protein family described in sequences databases have complex multi domain architecture with huge unstructured regions. Second, the new sequences selected through automatic methods contains relevant amount of sequence error (Yonghua et al., 2004[70]; Wen and Tan, 1996[68]). 

There are various methods which can be used to solve MSA problem such as the iterative (Mohsen et al., 2007[39]) classical, progressive algorithms (Kupis and Mandziuk, 2007[33]). All these algorithms are based on global or local alignment (Wei et al., 2013[66]; Changjin and Tewfik, 2009[8], Ankit and Huang, 2008[2]) techniques. The Global alignment technique, aids in making the sequences aligned from end to end points. Whereas, the local alignment technique first identifies a substring within a string and then tries to align it with the target string.

In general, local alignment is considered for sequence alignment but some time it creates problem because here in local alignment we have to deal with an additional challenge of identifying the regions of similarity. A dynamic programming based approach which are mostly used as the local and global alignment technique is the Smith-Waterman algorithm (Haoyue et al., 2009[22]) and Needleman-Wunsch algorithm (Needleman and Wunsch, 1970[43]). The dynamic programming (DP) (Zhimin and Zhong, 2013[72]) approach are considered to be good alignment option for not more than two sequences. Here, one thing is to be noted that MSA is a combinatorial problem (NP-hard) (Kececioglu and Starrett, 2004[30]) and when the number of sequences increases the computational effort becomes prohibitive. Feng and Doolittle (1987[15]) proposed a progressive alignment algorithm (tree-base algorithm), which uses the method of Needleman and Wunsch and for constructing an evolutionary tree (Bhattacharjee et al., 2006[4]) to know the relationship between sequences. The progressive alignment algorithms perform it operation through branching order of a guide tree and thus often get trapped to local optima (Naznin et al., 2012[42]). To avoid such kind of local optima it is suggested in the literatures to use either stochastic or iterative procedure (Mohsen et al., 2007[39]; Gotoh, 1982[19]). 

By referring to various literature studies (Devereux et al., 1984[11]; Jagadamba et al., 2011[27]; Nguyen and Yi, 2011[45]; Katoh et al., 2005[29]; Pei and Grishin, 2007[50]; Li et al., 2004[35], Ma et al., 2002[37]; Pearson, 2000[49]), it can be concluded that none of the existing algorithms were accurate enough to provide an optimal alignment for all the datasets. As a result, with the uses of iterative refinement strategies (Gotoh, 1982[19]), Hidden Markov Models (Eddy, 1998[13]) or Genetic Algorithms (Peng et al., 2011[51]) an iterative algorithms (Mohsen et al., 2007[39]) were developed to construct more reliable and efficient multiple alignments. Also, all these methods listed above have shown their superiority in aligning distantly related sequences for a variety of datasets (Blackshields et al., 2006[5]; Thompson et al., 1999[62]). However, some accuracy was degraded while considering the distantly related sequences. 

The above paragraph gives a clear indication that none of the method listed above can provide an accurate or meaningful alignment in all possible situations, irrespective of their advantages or disadvantages. Progressive alignment methods are known to be very fast and deterministic, but it suffers from a problem in which if any error occurs in the initial alignment and somehow gets propagated to other sequences than it cannot be corrected. However, this type of problem does not exist for iterative methods. In general, iterative methods are much slower in comparison to progressive methods and are used in a place where the best possible alignment is of prime importance and not the computational cost. 

Evolutionary algorithms such as the genetic algorithm, which are based on the natural selection processes, are used for implementing iterative methods. Such algorithms have an upper edge with respect to others in the sense that these algorithms are independent for any types of scoring function. This gives an independency that without much alteration to the alignments, different objective functions can easily be tasted. Also, evolutionary algorithms can give low-cost clusters and multi-core processors because of they can be easily parallelize to meets the current trend. 

In this study, genetic algorithms (Pengfei et al., 2010[52]) has been considered for experimental analysis. The main advantage of using GA for MSA problem is that it does not requires any particular source of algorithm to solve a given problem. Only, requirement for GA is the fitness function (Dongardive and Abraham, 2012[12]), for necessary analysis and evaluation of solutions. Because GA is an highly implicitly parallel technique therefore, it can be used to solve various large scale and real time problems such as the travelling sales man problem (Zhang and Wong, 1997[71]; Ulder et al., 1991[63]). For a sequences of smaller length it can be possible to do the alignment manually but sequences of larger length requires an algorithm for successful alignment. Progressive alignment technique such as the dynamic programming (DP) suffers from a problem of early convergence or local optima problem and hence cannot be used for alignment of larger sequences. Since, this research work is based on sequences of larger length (see Table 1(Tab. 1)) therefore approaches like GA is considered over DP.

Analyzing the importance of protein sequences in near future (Thompson et al., 2011[61]) provoked the author for considering MSA of protein sequences for this research work. Till date, sequence homology is considered to be the main method for predicting protein structure and function along with their evolutionary history (Kimura, 1980[31]). It has been observed that in the recent years, the tools (Gelly et al., 2011[16]) for MSA of protein sequences has improved. Various literature and related studied have confirmed that the further improvement in protein sequences can only be possible by combining sequence alignment with some know protein structures. A better performance of alignment of protein sequences can be excepted by proper utilizing the phylogenetic relationships among sequences (Cai et al., 2000[7]). 

Literature studies (Wong et al., 2000[69]; Taylor, 2000[58]; Razmara et al., 2009[54]; Mott, 2005[41]) says that there are still a number of challenges in aligning protein sequences. First, the misaligned or less aligned locally conserved regions within the sequences are major and foremost challenges in aligning protein sequences. Second, the misalignment of motif which is found in natively disordered regions. Third, the protein sequences which are found in various databases across the globe contain huge amount of alignment error (Loytynoja and Goldman, 2008[36]). 

On the basic of literature survey (Devereux et al., 1984[11]; Jagadamba et al., 2011[27]; Nguyen and Yi, 2011[45]; Razmara et al., 2009[54]; Mott, 2005[41]) and in order to test the feasibility of the proposed approach a comparison study were made between the proposed method and some of the existing methods such as the SAGA (Notredame and Higgins, 1996[46]), MSA-GA (Gondro and Kinghorn, 2007[18]), RBT-GA (Taheri and Zomaya, 2009[57]), CLUSTALX (Thompson et al., 1997[59]), CLUSTALW (Thompson et al., 1994[60]), HMMT (Eddy,1995[14]), PRRP (Gotoh, 1996[19]), PILEUP8 (Devereux et al., 1984[11]) and DIALI (Morgenstern et al., 1996[40]) by calculating the corresponding BAliscore. Some of these methods are iterative and some of these are progressive. Each of these methods has their own advantages and disadvantages in terms of speed, time, convergence, robustness and ability to align different lengths sequences etc. All such factors which promoted the author to select these different methods for the experimental study are mentioned in the paragraph that follows. 

SAGA, MSA-GA and RBT-GA are the GA based methods. The time complexity of SAGA is larger and are not suffers from the problem of local minima. RBT is an iterative algorithm for sequence alignment using a DP table. CLUSTALW can be seen as an example of progressive approach, and can be used to short out the local optimality problem for the progressive alignment approach. This is the most popular, accurate and practical method in the category of hierarchical methods. The widely used programs for MSA are CLUSTAL W and CLUSTAL X. They are very fast and easy to handle and are capable of aligning datasets of medium sized. The sequences so produced by these methods are of sufficient quality and not requires any manual editing or adjustment. HMMT is based on simulated annealing method. PRRP is a global alignment program which is based on a progressive and iterative approach. This approach is robust. PIMA (Smith and Smith, 1992[56]) uses a local dynamic programming to align only the most conserved motifs. DIANLIGN (Morgenstern et al., 1996[40]) uses a local alignment approach that construct MSA based on a segment to segment comparison rather than residue to residue comparison.

T-Coffee (Notredame et al; 2000[47]) method which was able to make very accurate alignments of very divergent proteins but only for small sets of sequences and therefore not considered for this experimental study. Also this method is often tapped at local minima. It also has a high computational cost with respect to other methods mentioned above. MAFFT (Katoh et al., 2005[29]) is very fast and can align sequences ranging from hundred to thousand. It is quite similar to CLUSTAL when it comes to alignment accuracy. But we have also not considered this method in the proposed research work, as the dataset and the fitness measure used by this algorithm is totally different than those used in this experimental approach.

The rest of the paper is organized as follow. The next section describes the relevant preliminaries on Alignment, Sequence alignment, MSA, GA, BAliBase and PAM Matrix, followed by the proposed approach section which describes the concepts underlying the research work. The experiments setups required in order to validate and observe the results are discussed in the next section. The second last section explains about the detailed results over different datasets. Finally, the concluding section presents the final consideration. 

## Preliminaries

This section provides a detail idea about the basic concept of the related terms used in the paper such as Alignment, Sequence Alignment, Multiple Sequence Alignment, GAP, BAliBase and PAM Matrix.

### Alignment 

The arrangement of two or more biological sequences in such a way that tells us at what point the sequences are similar and at what point they differ is known as alignment. An alignment is said to be the optimal one, if it has more similar sequences as compared to dissimilar sequences. 

### Sequence alignment 

Sequence alignment is a way of arranging the biological sequences so as to identify the region of similarity that may be a result of structural, functional, or evolutionary relationships between the sequences (Hicks et al., 2011[23]). In bioinformatics, the aligned sequences of DNA, RNA, or Protein are represented inside the matrix, in the form of rows. Gaps are inserted at some point in the sequences to achieve maximum similar character in a column.

It aims to infer clues about the unknown sequence by inferring biological characteristics of the matched sequence. One of the most challenging tasks in sequence alignment is its repetitive and time-consuming alignment matrix computations (Weiwei and Sanzheng, 2000[67]). 

### Multiple sequence alignment 

**Figure 1:**
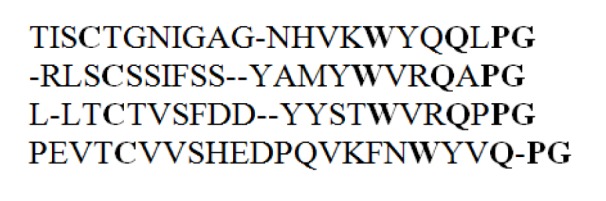
Example of a multiple sequence alignment

By referring to Figure 1(Fig. 1), we can define multiple sequence alignment (MSA) as the optimal alignment technique of three or more sequences with or without inserting gaps (Loytynoja and Goldman, 2008[36]). It plays an important role in sequence analysis and can also be used to judge and identify the similarity between DNA, RNA or protein sequences. With these features, MSA is proved as an important tool for prediction of function and/or structure (Layeb and Deneche, 2007[34]) of an unknown protein sequences. 

An MSA can be obtained by inserting gaps “-” at proper places such that no column in the sequences contains only gap character. Insertion of gaps will result in equal length sequences in the resulting alignment. 

Note 1: Consider an input string N1, N_2_.....N_p_ where a MSA maps them to some other string M_1_, M_2_....M_c_, where

1. |M_1_| = |M_2_| =....=|M_c_|

2. M_i_ by removing all “-” gap characters is equal to N_i._

3. None of the column contains only the gap character.

In MSA, there are various measures to evaluate alignment. 

### Gaps 

In order to have the best resulting alignment, gaps are permitted within the sequences along with a user defined mechanism for penalizing these gaps. Gaps are inserted between the residues so that identical or similar characters are aligned in successive columns. 

The values of gap penalties depend on the choice of matrix such as the PAM250 (Dayhoff et al., 1978[10]) (refer to PAM matrix section), PAM350 or the Substitution matrices such as BLOSUM which are used for sequence alignment of proteins. A Substitution matrix assigns a score for aligning any possible pair of residues and must balance their values. Adopting a high gap plenty scheme will restrict the appearance of gaps within the alignment. On the other hand, a too low gap plenty scheme will allow the gaps to appear everywhere in the alignment.

### Genetic algorithm 

Genetic algorithm is a type of iterative algorithms which allows an efficient and robust search. In the search process, a genetic algorithm starts with an initial state (population) in the solution space and in every search step, it produces a new and usually a better set of solutions. At each stage, GA moves forward towards producing a better solution which may led to minimize the change of getting trapped into a local extrema (Michalewicz, 1992[38]). Genetic algorithms are capable of handling large and complex scale problems (Jong, 1998[28]). Some applications of genetic algorithms for solving MSA problem can be found in (Goldberg, 1987[17]; Grefenstette and Fitzpatrick, 1985[20]; Holland,1975[25]; Hillsdale and Lawrence, 1987[24]; Buckles et al., 1990[6]). The references cited above, explain the GA approach and its ability to produce optimal solution for solving MSA problem of protein sequences. With addition to the above, there are various merits of genetic algorithms which can be utilized for prediction, alignment and classification of protein, DNA and RNA sequences and their structural and behavioral study (Dandekar and Argos, 1992[9]; Unger and Moult, 1993[64]; van Batenburg et al., 1995[65]). 

The major elements of genetic algorithm consists of representing a solution space, a fitness function, reproduction, crossover and mutation. In every step of GA operation, the genetic operators were applied to the solution space in order to produce new and better individuals for coming generations. A search may terminate when no further improvement is observed in the coming generation as compared to its previous one or when a predefined condition is met. 

### BAliBase 

BAliBase dataset is considered to be the standard dataset for alignment of protein sequences. It consists of variable lengths protein sequences which includes 218 sets of sequences taken from different sources. Here, the sequences are differentiated based on their similarity and structure in PDB database (Neshich et al., 1998[44]). To evaluate the quality of the obtained alignment, the BAliBase defined two sets of score namely SP Score and TC Score.

### PAM Matrix 

PAM which stands for point accepted mutation is used for the replacement of amino acid in the primary structure of protein. This statement will not involve any point mutation in the DNA of an organism. In general, silent mutation is not considered to be a point accepted mutation or lethal mutation.

PAM matrices encode the evolutionary change recorded at the amino acid level and are known as amino acid substitution matrices. The PAM matrix is constructed in such a way, that it can easily compare two sequences which are a specific number of PAM units apart. For example, the PAM120 score matrix is used to compare such sequences which are 120 PAM units apart.

## Proposed Approach

This section detailed about the proposed approach which is based on various parameters and are described below.

### Representation and initial generation 

In the proposed approach, the population is initially randomly generated at first. Based on the largest sequence size, the initially generated population is filled with a random gap sign to make the initially generated sequences equals to the largest sequence in the set. Also, the gaps are inserted within the sequences keeping in mind that the total size of the gap does not exceed 25 % total length of the largest sequence. After the initialization process is over, the solution set is combined and then mutated for further operation so as to produce new individuals with a defined number of generations (iterations), which is 50 for this experimental study.

### Scoring function 

In this section, a formal definition of the sum-of-pairs of multiple sequence alignment is introduced which is used as a tool to calculate fitness.

Proteins or genes perform the same function because of their similar sequences. DNA stores all genetic information of an organism while the Proteins act as the building blocks for all the cells. There are total 20 linear chain of amino acid for protein which are denoted as:

E,P,A,C,G,Q,V,M,T,R,K,W,Y,D,N,H,S,F,L and I. 

Similarly, DNA is represented by four nucleotides namely A, C, G, T. Therefore, in general we usually represent protein and DNA sequence through a string of small alphabetical letters. Here, for every protein sequences the sum of scores based on their fitness functions is calculated. Obtaining a best alignment is dependent upon the scoring criteria followed in order to build that alignment. Therefore, a scoring matrix know as the sum of pair score and the match column score is adopted to calculate the alignment scores between two characters within a column (Otman et al., 2012[48]).

For the experiment, the gap penalty is taken as:

J={E,P,A,C,G,Q,V,M,T,R,K,W,Y,D,N,H,S,F,L and I }


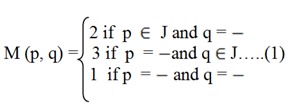


Equation (1) suggests that

If p Є J and q = - then the gap penalty is taken as 2.

If p = - and q Є J then the gap penalty is taken as 3.

And if, p = - and q = - then the gap penalty will be taken as 1.

If p Є J and q Є J then use PAM 250 matrix. In case of match occurs refer to PAM 250 (Dayhoff et al., 1978[10]) matrix available online.

Here, the gap penalty stated in equation 1 is user defined and will remain fix for a complete set of experiment. Here, the penalty for gap extension and opening is not same.

### Fitness evaluation 

To judge the quality of different alignments based on their scores, a fitness function is proposed which is defined in equation 2.

For scoring purpose, PAM 250 Matrix has been used as a scoring matrix to calculate score between different alignments. 

In the experiment the fitness is calculated as:-


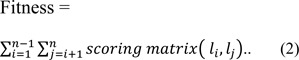


Where, 

n = number of sequences, *l_i_* = first sequence, *l_j_* = second sequence 

The score for each column in an alignment is scored by summing the score of each pair of symbols. The overall alignment score is then calculated by using equation 1 and 2, which should be best possible maximum value.

### Selection strategies description 

The selection methods used in this research is here under:

Sorting of individuals is done in the mating pool according to their fitness and then every two best individuals are selected for crossover.

### Child generation 

In order to generate a child population of 100 individuals in every generation, two genetic operators namely Crossover and Mutation have been considered for the experimental study, which are described below in details.

### Crossover

Crossover operation is performed over the two strings of biological sequences by randomly selecting a cutting point and swapping the string from that point with a predefined probability.

### Crossover operator I

**Figure 2:**
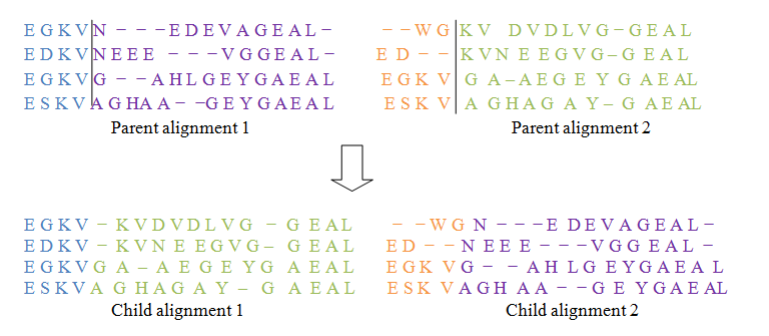
One point crossover I

As shown in Figure 2(Fig. 2), this operator first chooses a column randomly in the parent alignments and defines a cut point there. Then by interchanging the different parts of parents it form two new offsprings, also known as Childs. For doing this type of operation gaps may be added to the resulting offsprings. 

### Crossover operator II

**Figure 3:**
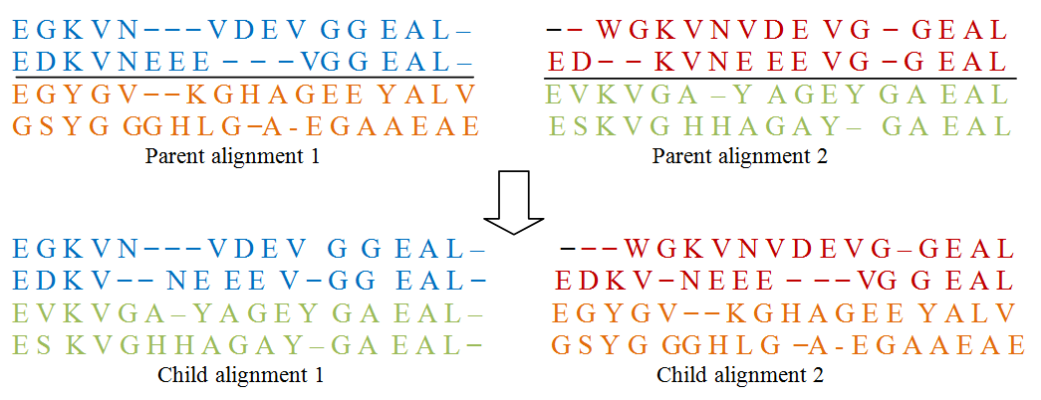
One point crossover II

Same as in I and as described in Figure 3(Fig. 3), this operator also chooses a point in the given parent alignment and cuts the alignment from that point. Again by swapping different parts of parent alignment it produces child alignment by inserting gaps at required positions. 

### Mutation 

After crossover, the strings are moved for mutation (Otman et al., 2012[48]). Mutation prevents the algorithm to be trapped in a local minimum. It distributes the genetic information randomly among other individuals and helps to recover the lost genetic materials. Mutation operation involves randomly flipping of few bits in a chromosome. For example, the string 00100100 might be mutated in its second position to yield 01100100. Mutation operation can happen with very small probability at each bit position in a string.

The mutation operators are exclusively being used in this experimental study. As we all know the mutation operators are used for regaining the lost genetic operator therefore, in this study the mutation operators are used with a very least probability of 0.01 to improve the overall quality of the sequences or for getting a good aligned sequences. In this approach, when the sequences are subjected for mutation operation, then flipping or swapping of nucleotides is being done within the sequences so as to improve the overall score of the alignment which ultimately results in high quality solutions. Flipping or swapping of nucleotides and placing it to somewhere else in the sequences may results in improving the alignment quality of the sequences. As matching of nucleotides in the same row or column is possible by swapping or flipping of nucleotides. All the defined mutations operators are used one by one to check which of these operators gives a better result in terms of score. The operator which give the highest results is considered and rest are declined for that particular sequences (dataset). 

All the different mutation operators defined were selected at a random basic to solve a given set of problem with a very small probability of 0.01. Here, in the proposed approach when one of the randomly selected mutation operator fails to given an optimal results, then a different mutation operators from the defined one is selected and applied to solve the given problem. All the proposed mutation operators for the experimental analysis are described below.

### Exchange mutation operator

**Figure 4:**
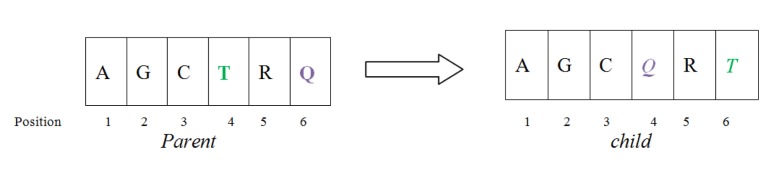
Exchange Mutation operator

This mutation operator is explained in Figure 4(Fig. 4) in which, the position of two nucleotide (position 4 and 6) are exchanged which are randomly chosen.

### Reverse mutation operator

**Figure 5:**
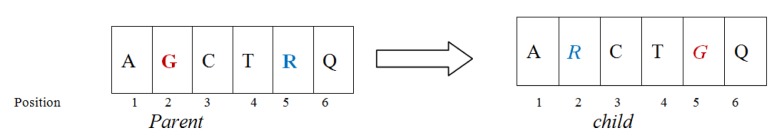
Reverse Mutation operator

This mutation operator is clearly illustrated in Figure 5(Fig. 5). Here, a sequence S has taken which is limited by two randomly chosen position 2 and 5. The order of nucleotide in this sequence will be reversed in the same order as covered in the previous operation.

### Position mutation operator

**Figure 6:**
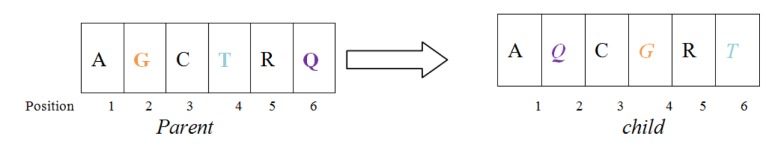
Position mutation operator

In this mutation operator, Three nucleotide were randomly chosen which shall take the different positions not necessarily successive 2 < 4 < 6. The nucleotide who is currently at the position of 2 will take the position of 4 and one who was at 4 will take the position 6 and again the nucleotide holding this position currently will occupy the position of 2. Figure 6(Fig. 6) demonstrate the processes discussed above. 

### Inverse mutation operator

**Figure 7:**
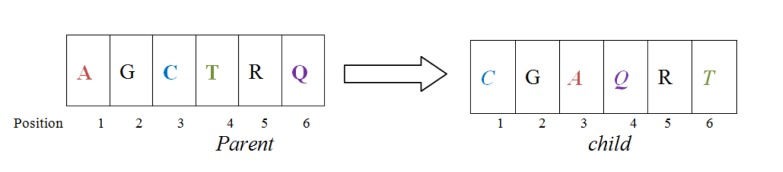
Inverse mutation operator

In Figure 7(Fig. 7), two sections of nucleotide were made by dividing the chromosomes into two sections. All nucleotide in each section are copied and are placed inversely in the same section of a child.

### New generation

For the coming generation, a 60-40 % selection scheme of parent - child combination based on their fitness score is implemented. It means that for the coming generation 60 % of the parent and 40 % of the child population will be used to produce the next population.

Other combinations such as 40-60 % or the 50-50 % parent - child population has also been considered but, these strategies has not shown any impact in improving the overall quality of the solution and hence not been considered. Also, 100 % crossover and 100 % mutation operation were considered along with 40-60 % or the 50-50 % parent - child population, but these combinations were not able to bring any changes in the overall quality of the solutions so produced. Table 2(Tab. 2) explain the parameter analysis based on 60-40 %, 40-60 % and 50-50 % parent - child combination along with the results 100 % crossover and 100 % mutation operation. It can be observed that the time taken to calculate 60-40 % selection scheme of parent - child combination is least as compared to 40-60 % or the 50-50 % parent- child combination or any other scheme discussed in Table 2(Tab. 2). The average computation time mentioned in Table 2(Tab. 2) is the time taken to perform the experiments for each datasets. However, no comparative study of computation time with different methods mention in Tables 3(Tab. 3), 4(Tab. 4) and 5(Tab. 5) were made. As, there is no such data available in the literature study for such type of comparison.

### Termination condition

The termination conditions used for the experiment are as follows: 

In the experimental study, we have tasted the results on maximum 50 iterations (generations), and hence made the experiment to be terminated after reaching 50 iterations, as there is negligible amount of improvement in the alignment quality.

## Algorithm for the Proposed Method

Step 1 : Population initialization x_1_,x_2_,...,x_n._

Step 2 : Column(N) = 1.2 x n_max. _Gaps (-) may be placed in the sequences for proper alignment. 

Step 3 : Compute fitness.

Step 4 : Select individuals for genetic operations. Two different genetic operators mainly crossover and mutation is used with probability of 0.8 % and 0.01 %.

Step 5 : Do crossover operation by randomly choosing any one of the defined crossover operator. 

Step 6 : Randomly choose and apply all of the defined mutation operator one by one.

Step 7 : Check all the four solution quality, and choose the one who is the best among all four solutions in terms of scores.

Step 8: New population generated and fitness evaluated.

Step 9 : Stop if sufficient solution quality or max search terms reached, which is 50 iteration.

## Experimental Set Up

This section gives an overview of the parameters and the systems components used for the experiment.

### Parameters setting for the experiment

The population size was established to 100 individuals and the maximum number of generations (iteration) was 50 with a crossover probability of 0.8 %, mutation rate of 0.01 %. The scoring matrix used for the experiment is PAM 250 for each Protein sequences. Here, the population size of 100 suggests that for each generation/iteration the algorithm runs for producing 100 childs with the help of proposed genetic operators. And among these 100 childs so produced, the two best childs based on their scores are selected to be the parents for the next generation.

### System components

The main objective of this research work is to observe the role of proposed crossover and mutation operators in solving MSA problem of protein sequences in terms of quality and scores of the sequence aligned. Here, quality of an aligned sequence is judged by the scores it obtains after successfully aligning. In this study, the experiments for the proposed approach have been performed using genetic algorithm with C programming on an Intel Core 2 Duo processor having 2.53 GHz CPU with 2 GB RAM running on the Linux platform.

## Results and Discussion

In this section, the experimental methodology followed in this work is detailed. Moreover, results obtained with the proposed method are presented and discussed. 

For all the tests, the different crossover and mutation operators are randomly chosen with equal probability of selection within each generation. To test the proposed approach, the experiments are carried out with different datasets (ref. 1, ref. 2 and ref. 3) of different lengths from the BAliBase database (refer Table 1(Tab. 1)). The author used these datasets for the experimental study because of their performance with other related algorithm, which are gained by referring various literature studies (Devereux et al., 1984[11]; Jagadamba et al., 2011[27]; Nguyen and Yi, 2011[45]; Razmara et al., 2009[54]; Mott, 2005[41]). As stated earlier, for every experiment the alignments were performed with the proposed method and were compared with the methods described in the literature study stated earlier. 

For evolution of the proposed approach, the algorithm were executed for 50 independent run (iterations) for 30 datasets (some of all datasets in Table 3(Tab. 3), 4(Tab. 4) and 5(Tab. 5)) and then the best, average and the worst score were calculated. Table 1(Tab. 1) indicates the best, average and the worst score over different datasets with their corresponding BAliscores. As, the fitness score depends upon the level of similarity among the residue in the sequences therefore, the scores can be either positive or negative. Here, one point is to be noted that if the residues among the comparable sequences are similar, then small numbers of gaps (“-”) are needed to make the sequences aligned properly. On the other hand, if the majority of the residues are dissimilar then a large number of gaps are needed for necessary sequence alignment.

**Figure 8:**
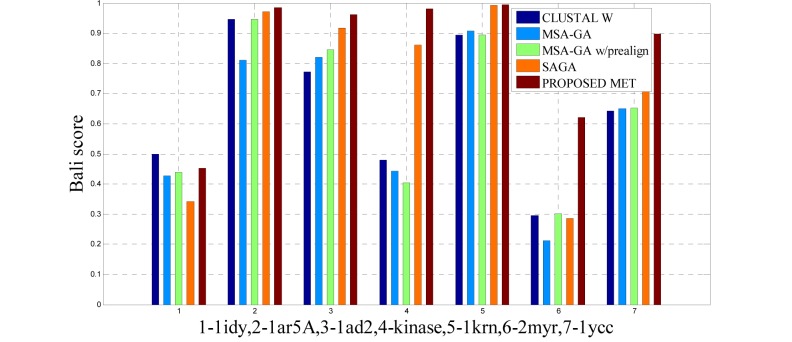
Bar graph comparison result of scores between proposed and other methods over Ref. 1

**Figure 9:**
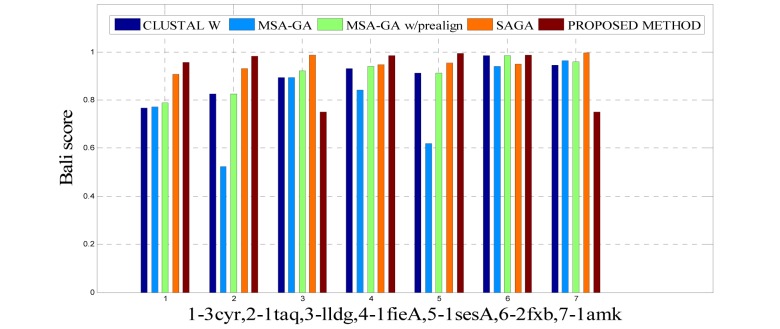
Bar graph comparison result of scores between proposed and other methods over Ref. 1

**Figure 10:**
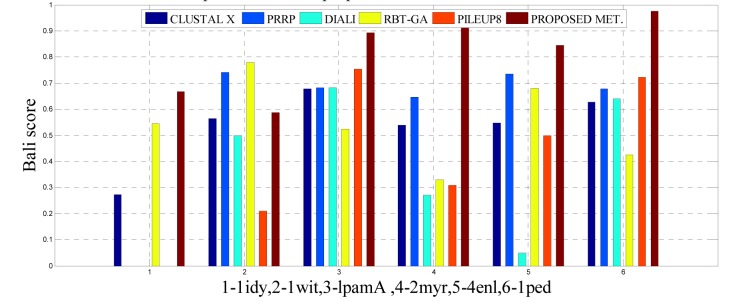
Bar graph comparison result of scores between proposed and other methods over Ref. 3

**Figure 11:**
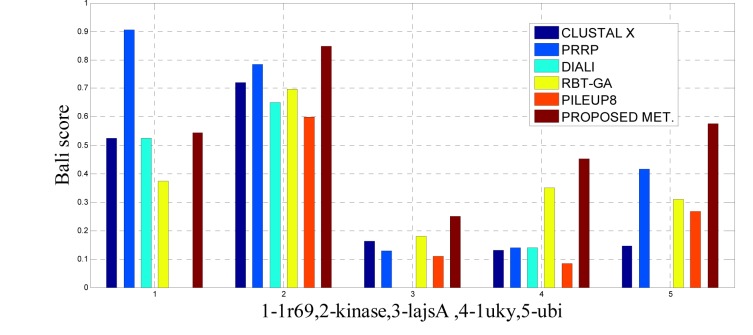
Bar graph comparison result of scores between proposed and other methods over Ref. 3

**Figure 12:**
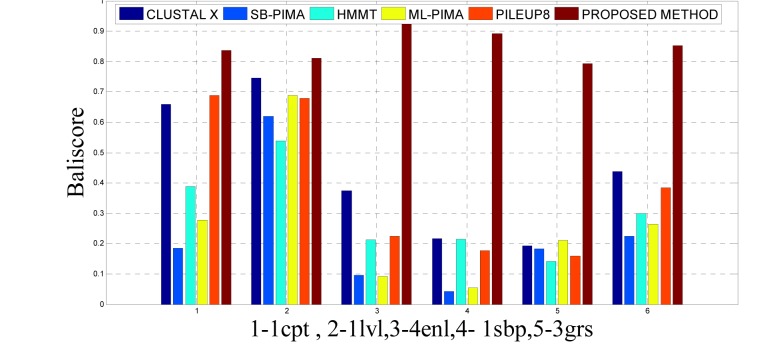
Bar graph comparison result of scores between proposed and other methods over Ref. 2

**Figure 13:**
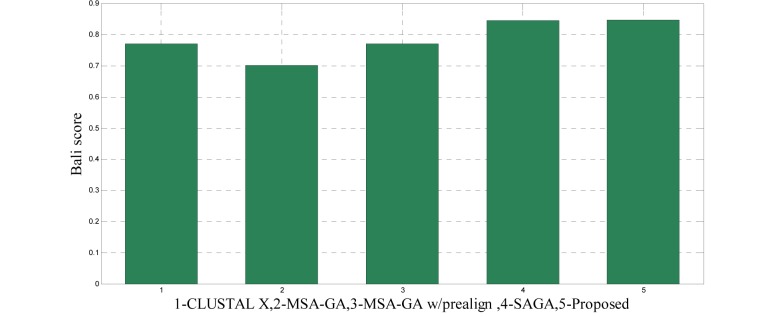
Average score comparison between proposed and other methods over Ref. 1

**Figure 14:**
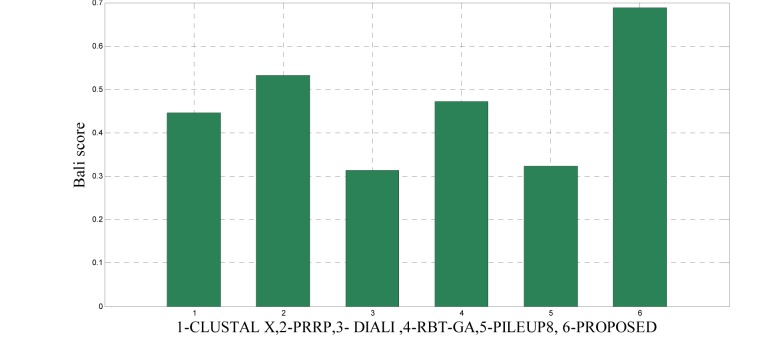
Average score comparison between proposed and other methods over Ref. 3

**Figure 15:**
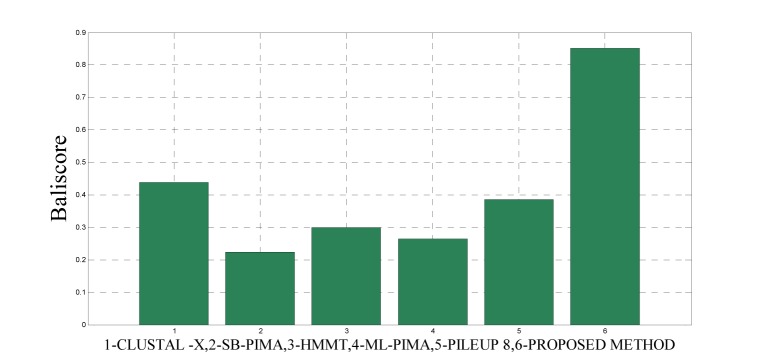
Average score comparison between proposed and other methods over Ref. 2

To analyze the quality and accuracy of solutions produced by the proposed approach, we have considered a BAliscore, which is an open source program of the BAliBase benchmark. BAliBase scores a solution (multiple sequence alignment) between 0.0 and 1.0. A score of 1.0 indicates that the solution is same or identical to that of manually created reference alignment. Unfortunately, with the proposed approach we are unable to get a score equals to 1(see Tables 3(Tab. 3), 4(Tab. 4) and 5(Tab. 5)). If the score is 0 then it indicates that nothing matches to the reference alignment. This can be observed with some of the datasets in Table 4(Tab. 4) (reference 3).The score between 0 and 1 indicates that some part matches with the reference alignment. The scores which are closer to 1, gives a better alignment for a given dataset. A comparison over different datasets with different methods is being made in Tables 3(Tab. 3), 4(Tab. 4) and 5(Tab. 5). By referring to these tables, we can conclude that the proposed method solution is much more efficient than other methods in terms of scores as indicated in the tables. Figures 8(Fig. 8), 9(Fig. 9), 10(Fig. 10), 11(Fig. 11) and 12(Fig. 12) shows comparative results between the proposed and the other methods discussed in the literature review earlier. Figures 13(Fig. 13), 14(Fig. 14) and 15(Fig. 15) indicates about the average scores comparison among different methods and gives a clear indication about the superiority of the proposed approach over the others.

In order to evaluate the overall performance of the proposed method, the average score of all test cases were evaluated (bottom of Tables 3(Tab. 3), 4(Tab. 4) and 5(Tab. 5)). The average score suggest that the proposed method approach is better among all other methods that are considered. The score is calculated considering the standard BAliBase dataset. The bold faced data`s in the tables indicates the best scores among the methods.

### Performance of the proposed method with Ref. 1 

The 14 datasets of reference 1 shown in Table 3(Tab. 3) are of different lengths and sequences (refer Table 1(Tab. 1)). In order to compare the proposed method with respect to BAliscore, the proposed approach were compared with that of CLUSTAL W,MSA-GA, MSA-GA w/ prealign and SAGA. From comparison in Figure 8(Fig. 8) and 9(Fig. 9), it can be seen that out of 14 test cases, the proposed method has successfully overcome other methods solutions in 11 test cases and in three test cases, the proposed method solution were very close to the best.

### Performance of the proposed method with Ref. 3 

In this experimental study, eleven test cases were considered from references 3, again out of 11 test cases the proposed method shows better solution for 9 test cases. Only, RBT-GA for 1wit dataset and PRRP for 1r69 dataset shows better performance than the proposed method. The results are provided in Table 4(Tab. 4) and Figure 10(Fig. 10), 11(Fig. 11) and 14(Fig. 14).

### Performance of the proposed method with Ref. 2

As detailed in Table 5(Tab. 5) and Figure 12(Fig. 12) and 15(Fig. 15), five dataset from ref. 2 were considered for evaluating the proposed approach with some standard methods such as the CLUSTAL X, SB-PIMA, HMMT, ML-PIMA and PILEUP8. Experiment on benchmarks (BAliBase 2.0) were conducted and observed that the proposed method technique is much efficient than the other compared ones.

### Performance characterization of proposed algorithm

Two different components namely the proposed genetic operators and random population initialization plays an important role in making the performance of the proposed algorithm better than other algorithms. Two different set of experiments have been designed in order to investigate the performance of the proposed algorithm. In the first case, a different approach for population initialization is adopted (different than the proposed scheme). Here, the proposed algorithm was made to run with a randomly generated population, constructed with the help of guide tree. In the second case, a hill climbing approach (Huiying and Zheng, 2013[26]) (for searching instead of proposed algorithm) has been used, which starts from the same random initial population used in this work. The fitness evaluation scheme will remain the same as discussed in the proposed approach section. A total of fifteen BAliBase datasets (five from each ref 1, 2 and 3) is considered for the experiments. Each datasets was made to run with the proposed algorithm (with two different cases stated above) for fifty iterations. Based on the BAliBase score the best scores were recorded, and it was analyzed that the proposed algorithm with random initial population generation outperformed the guide tree initial generation technique for all the datasets. The average improvement of 9.72 % was recorded with randomly generated population. Similarly, with hill climbing approach the proposed algorithm was recorded with an average improvement of 7.23 %. Thus, with the above discussions we can say that the proposed algorithm with randomly generated initial population and proposed genetic operator is superior to other algorithm in terms of performances. The detail experimental results are available in Table 6(Tab. 6).

## Conclusion

As we all know that the multiple sequence alignment is a known problem in bioinformatics, but still MSA remains a challenging task to explore. The arrangement of molecular sequences within an alignment to find similarities and differences among them is not an easy task, due to the complex size of the sequences and the search space. Because of the ability to handle complex scale problems, genetic algorithm is used as a genuine solution for the multiple sequence alignment problem. In this paper, a novel approach has been developed, which uses genetic algorithm for performing multiple sequence alignment. The motive of the study reported in this paper is to judge the efficiency of the proposed approach by comparing it with different algorithm over standard datasets. In order to evaluate the efficiency and feasibility of the proposed approach, a benchmark datasets from BAliBase 2.0 is considered, because most of the methods discussed in this paper uses BaliBase datasets to access the quality of the multiple sequence alignments. When compared to other methods listed in (Notredame and Higgins,1996[46]; Gondro and Kinghorn, 2007[18]; Taheri and Zomaya, 2009[57]; Thompson et al., 1997[60]; Eddy, 1995[14]; Gotoh, 1996[19]; Devereux et al., 1984[11]; Morgenstern et al., 1996[40]), the proposed method improves the overall quality of the alignment. The experimental result provides a better scope for multiple sequences alignment, as there is an increase in the alignment quality, which can be observed by the scores of different datasets. It was also observed that the proposed method solution gives some unsatisfied results in some test cases. By the above discussions, we can easily conclude that the innovative approach adopted in this paper gives a better and improved result when compared with other methods in most of the testcases.

## Figures and Tables

**Table 1:**
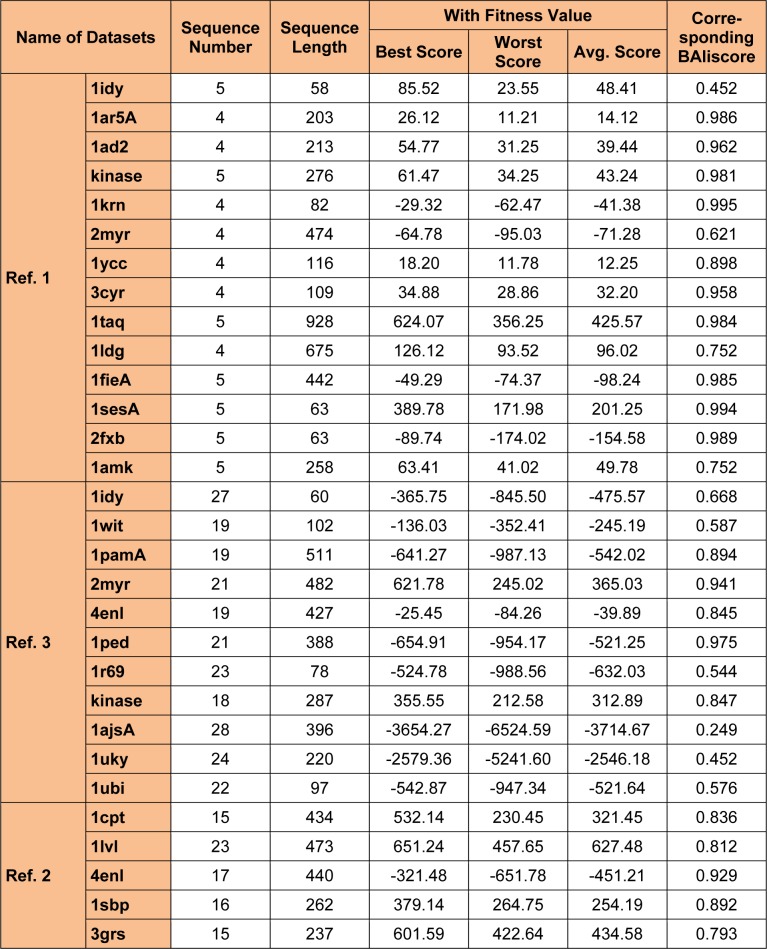
Summary of the test results of proposed method

**Table 2:**
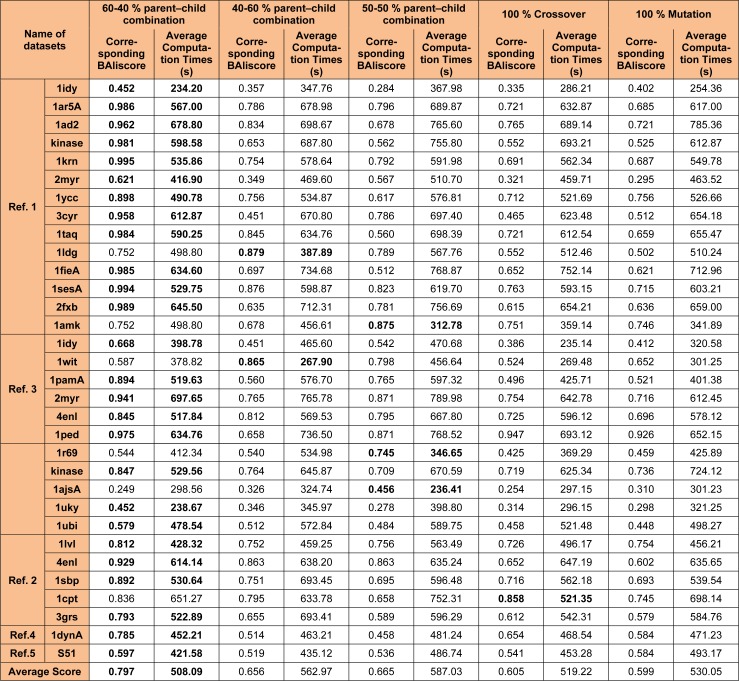
Average Computation Times(s) comparison over Ref. 1, 2, 3, 4 and 5

**Table 3:**
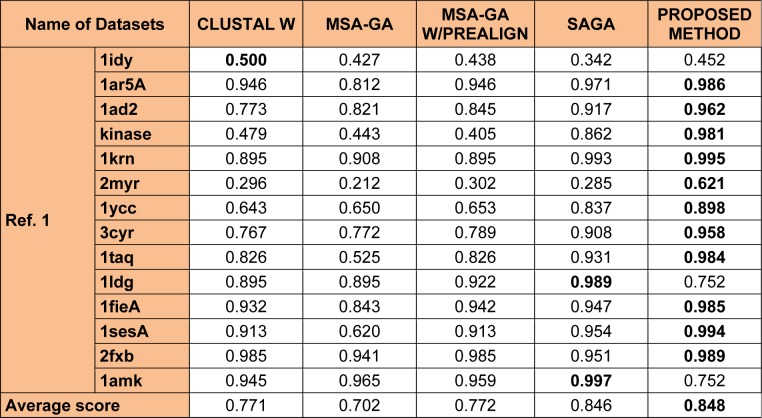
Experimental results with Ref. 1 datasets of BAliBase 2.0

**Table 4:**
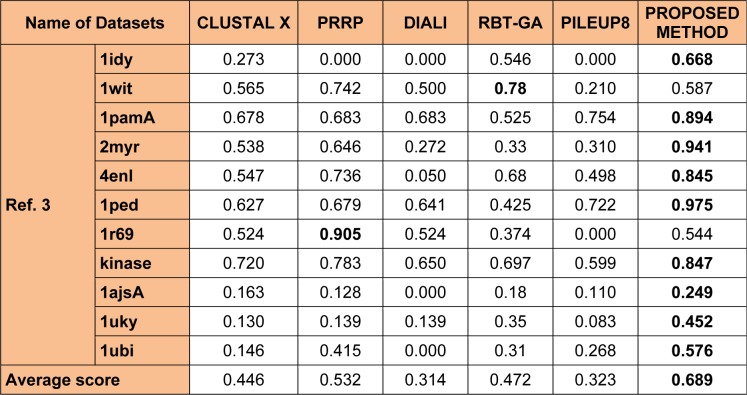
Experimental results with Ref. 3 datasets of BAliBase 2.0

**Table 5:**
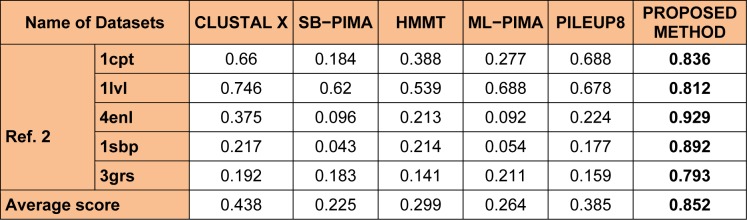
Experimental results with Ref. 2 datasets of BAliBase 2.0

**Table 6:**
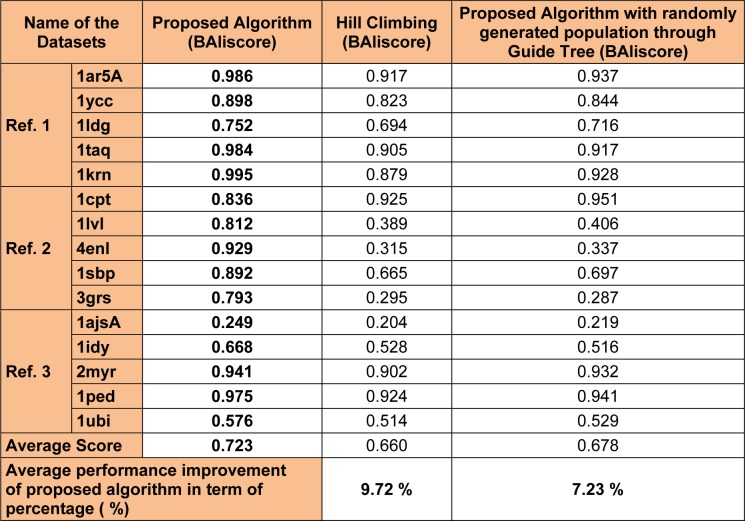
Performance evaluation of the proposed algorithm with hill climbing approach and randomly generated population through guide tree
